# Mental Health and Cognitive Development in Symptomatic Children and Adolescents Scoring High on Habitual Snoring: Role of Obesity and Allergy

**DOI:** 10.3390/children10071183

**Published:** 2023-07-07

**Authors:** Marco Zaffanello, Angelo Pietrobelli, Leonardo Zoccante, Giuliana Ferrante, Laura Tenero, Michele Piazza, Marco Luigi Ciceri, Luana Nosetti, Giorgio Piacentini

**Affiliations:** 1 Department of Surgery, Dentistry, Pediatrics and Gynecology, University of Verona, 37100 Verona, Italy; angelo.pietrobelli@univr.it (A.P.); giuliana.ferrante@univr.it (G.F.); laura.tenero@aovr.veneto.it (L.T.); michele.piazza@univr.it (M.P.); giorgio.piacentini@univr.it (G.P.); 2 Child and Adolescent Neuropsychiatry Unit, Maternal-Child Integrated Care Department, Integrated University Hospital of Verona, 37126 Verona, Italy; leonardo.zoccante@aovr.veneto.it (L.Z.); marco.ciceri@aulss9.veneto.it (M.L.C.); 3 Pediatric Sleep Disorders Center, Division of Pediatrics, Filippo Del Ponte Hospital, Insubria University, 21100 Varese, Italy; luana.nosetti@uninsubria.it

**Keywords:** adolescent, children, home respiratory polygraphy, obstructive sleep apnea–hypopnea index, oxygen desaturation index, questionnaires, sleep-disordered breathing

## Abstract

Background: Obstructive sleep apnea can have a negative impact on children’s and adolescents’ neurocognitive abilities and hinder their academic and adaptive progress in academic, social, and/or behavioral dimensions. In this retrospective cross-sectional study, we investigated the influence of body weight conditions and allergy status on long-term mental health, cognitive development, and quality of life in children and adolescents who snored. Methods: The study sample included 47 subjects (age range 4.1 to 15.3 years) who exhibited high levels of snoring and underwent home-based polysomnography between 2015 and 2019. Follow-up assessments (3 years on average between baseline and follow-up) entailed phone interviews with the subject’s parents/caregivers who completed three validated questionnaires investigating sleep, quality of life, and parental ratings. Results: We found a correlation between age at diagnosis and being retrospectively overweight and high levels of snoring. In addition to a higher risk of developing emotional symptoms (8.2% increase in retrospective overweight status for each unit increase in the emotional score at follow-up) and oppositional behavior (9% increase in retrospective overweight status for each unit of oppositional T points at follow-up), we also noted reduced long-term social symptoms (11% decrease in retrospective overweight status for each unit increase in the social score at follow-up) and cognitive symptoms (10.6% decrease in retrospective overweight status for each unit increase in the cognitive score at follow-up), as well as a 6.1% increase in retrospective allergy status for each unit increase in academic performance at follow-up. Conclusions: Snoring can have negative impacts on mental health and cognitive development in the long term. Early detection and intervention for neuropsychological disorders is important in children and adolescents who score high on snoring. In the long term, the effects of snoring on neuropsychological disorders may vary based on previous body weight and allergy status.

## 1. Introduction

The prevalence of obstructive sleep apnea syndrome (OSAS) in children varies depending on the population studied and the diagnostic criteria used. It is estimated that OSAS affects approximately 1% to 5% of children [1]. The percentage of individuals under the age of 18 who report regular heavy snoring ranges between 8% and 12%. Initial studies have estimated the prevalence of pediatric OSAS to be between 1% and 3% [2]. Sleep-disordered breathing (SDB) can lead to morbidities of the central nervous system (CNS), cardiovascular [3] and metabolic systems, body growth [4], and diminished quality of life (QoL) [5]. There is also evidence for an association between OSA and hyperactivity, inattentive behaviors, cognitive deficits, hyperactivity, and aggressive behaviors, albeit to a lesser extent in children who habitually snore [5]. OSA can have a negative effect on neurocognitive and neurological development, hindering a child’s academic and learning abilities [6]. Furthermore, SDB symptoms may adversely affect academic performance in children with overweight/obesity [7]. Behavioral functioning was noted to be worse in children originally diagnosed with SDB than controls. Nevertheless, the long-term cognitive and behavioral outcomes were found to be minimally affected by the resolution of SDB in preschool-aged children [8]. Accordingly, there is an ongoing search for clinically useful tools that can identify children at risk for developing cognitive and behavioral deficits [9].

Polysomnography (PSG), a multi-parameter sleep study performed overnight, is the gold standard for diagnosing SDB [10], while simpler tools for OSA diagnosis and screening have been developed and approved for settings with limited resources [11]. For example, questionnaires can be administered [12,13] to screen for SDB and evaluate the neuropsychological impact of OSA on children [14].

Childhood obesity is the second leading cause of snoring [15,16] and children who habitually snore are often noted to have an allergy [17]. Pediatric obesity remains an issue of global concern, affecting approximately 17% of children and adolescents in the United States [18]. In the short term, overweight children are more likely to suffer from depression, anxiety, low self-esteem, and a range of emotional and behavioral disorders [19]. Obesity in children with SDB during late childhood and adolescence is correlated with behavioral functioning, particularly inattention and learning difficulties, which ensues functional difficulties at school [20].

The increasing prevalence of allergies among children raises concern. Allergic diseases in children have significantly increased in recent years and now affect up to 35% of children [21]. Allergic rhinitis, for instance, affects approximately 400 million people worldwide [22,23]. The prevalence of wheezing in the preschool population was 23.7%. Among the children included in a study, 13.7% were classified as overweight and 5.7% as obese [24]. Allergic rhinitis appears to be present in 35% of children with primary snoring and in 6% with OSA [25]. Rhinitis can seriously disturb sleep in patients with OSA [26] and is a recognized risk factor for habitual snoring and for OSA in children [25].

Cognitive functions have been found to be affected during the pollen season in children with allergy, and the more symptoms an allergic child has, the longer the reaction time on cognitive tests [27].

Determining the differences between obese and normal-weight individuals, as well as between allergic and non-allergic children and adolescents who exhibit high levels of snoring, and understanding their long-term neuropsychological outcomes, remains a challenge. We hypothesize that overweight and allergy status have a significant impact on the long-term neuropsychological outcomes in children and adolescents who snore, with higher rates of adverse outcomes observed in those with overweight and allergies compared to those without these conditions. To address this issue, we conducted assessments at two distinct time points. The first assessment occurred during the retrospective enrollment of children, while the second assessment served as a follow-up.

## 2. Materials and Methods

In our retrospective cohort study, we recruited children and adolescents with a history of snoring. The study sample included both male and female Caucasian subjects who underwent cardiorespiratory PSG between 2015 and 2019 at the Department of Pediatrics, University of Verona, Italy. Recruitment was based on the availability of retrospectively collected data and the voluntary participation of the children’s parents/caregivers. Children and adolescents who retrospectively had incomplete or missing PSG recordings lasting less than 6 h, or who had comorbidities, such as neurological or neuromuscular diseases, genetic syndromes, or neuropsychiatric syndromes, were not included in the study. The protocol for clinical studies was approved by the ethics committee of our Integrated University Hospital (CESC601; 12 August 2015) Informed consent for the scientific use of data was obtained from the parents or guardians of the subjects.

### 2.1. Study Population

**Figure 1:**
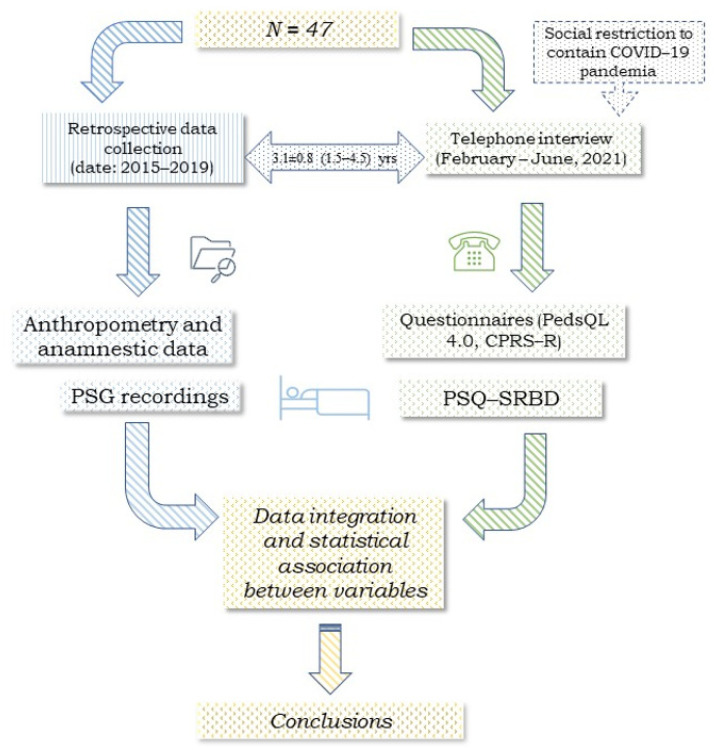
Flow chart of this retrospective cross-sectional study involving 47 children and adolescents. Legend: pediatric sleep questionnaire (PSQ-SRBD), the pediatric quality of life inventory (PedsQL 4.0), and the short version of Conners’ parent rating scale-revised (CPRS-R).

Figure 1 presents the study chart divided into two sections that illustrate the retrospective collection of clinical and medical history and the results of at-home overnight PSG recording, and the collection of telephone questionnaire responses (February to June 2021), respectively. The study sample included 47 children and adolescents with a history of snoring and had undergone at-home overnight PSG between 2015 and 2019 (Figure 1). At follow-up (February to June 2021), they were contacted via phone and they agreed to participate in the survey. The children’s or adolescents’ parents/caregivers were also asked whether the subjects also had a history of respiratory allergies.

### 2.2. Anthropometry

We retrospectively collected the height and weight of the enrolled subjects (Figure 1). They were measured using a precision medical scale (WUNDER C201, Wunder Sa.Bi. srl, Milan, Italy) and a telescopic stadiometer. Trained healthcare professionals conducted the measurements on lightly dressed subjects without shoes. Weight was recorded with an accuracy of 0.1 kg, and height with an accuracy of 0.1 cm. To calculate body mass index (BMI), BMI percentiles, and BMI z-scores, we utilized an online tool (http://www.bcm.edu/bodycomplab/BMIapp/BMI-calculator-kids.html, accessed on 30 June 2021) based on CDC growth charts for children and adolescents aged 2–19 years (https://www.cdc.gov/healthyweight/bmi/calculator.html, accessed on 30 June 2021). The subjects were categorized as underweight/normal weight when the BMI z-score was ≤0.99 and overweight/obese when it was ≥1.

### 2.3. Respiratory Polysomnography

We retrospectively collected the data of PSG recordings (Figure 1). The overnight PSG recordings were made using a portable ambulatory device (SOM-NOscreenTM PSG, SOMNOmedics GmbH, Randersacker, Germany) at the subjects’ home. The device continuously monitors physiological parameters, including nasal airflow through a cannula, respiratory movements of the chest and abdomen by means of belts, and oxygen saturation (SpO_2_) by heart rate, body position, and respiratory sounds, as previously described [14]. The children’s or adolescents’ parents/caregivers were trained to correctly use the device and taught to record the diary entries for the subjects’ bedtime, nighttime awakenings, and the child’s or adolescents’ wake-up time in the morning. The recordings were analyzed using DOMINO software (Somnomedics v. 2.6.0) by one of the investigators (MZ). MZ has experience in standardized reading and in continuous training, and follows recognized diagnostic criteria and guidelines for the interpretation of PSG recordings. The estimated total sleep time (eTST) was calculated [28]. Respiratory events were assessed following the guidelines established by the American Academy of Sleep Medicine [10]. The obstructive apnea–hypopnea index (oAHI) was defined as the sum of obstructive apneas, mixed apneas, and hypopneas divided by the eTST [28]. The ODI3% (oxygen desaturation index ≤3%) was determined by dividing the total number of desaturations by the eTST (events per hour). The average and minimum SpO_2_ (%) were computed automatically. Additionally, snoring events were assessed as a percentage of the eTST in the comprehensive overnight recording.

### 2.4. Telephone Interviews

At follow-up, the parents/caregivers of the children or adolescents were contacted via phone between February and June 2021 (Figure 1). Those who consented to participate shared their medical background, which included details about the child’s respiratory allergies and any past adenoid or tonsil surgeries that occurred after the examination. The parents/caregivers completed three validated questionnaires: the 22-item abbreviated version of the Pediatric Sleep Questionnaire (PSQ-SRBD), the Pediatric Quality of Life Inventory (PedsQL 4.0), and the abbreviated version of Conners’ Parent Rating Scale-Revised (CPRS-R).

### 2.5. Questionnaires

#### 2.5.1. Pediatric Sleep Questionnaire

The PSQ is employed as a screening tool for obstructive sleep apnea syndrome (OSAS) and to evaluate the quality of life of the children and adolescents [29,30]. It consists of 22 items with a “yes,” “no,” or “do not know” response. The overall score is calculated from the percentage of affirmative (“yes”) responses. A score is considered significant if the number of positive responses exceeds 33% of the total.

#### 2.5.2. Pediatric Quality of Life Inventory

The PedsQL is used to assess the quality of life in children and adolescents between the ages of 2 and 18 years [31,32]. It has been validated in Italian [32] and is age-related, with items categorized by age. The questionnaire consists of 23 items that investigate four domains: physical health and activity (8 items), emotions (5 items), social relationships (5 items), and school (5 items). The first 8 items pertain to physical health, while the remaining 15 investigate psychosocial health. Parents/caregivers are asked to reflect on the child’s and adolescent’s life in the month up to questionnaire administration [33,34]. A higher score corresponds to a better quality of life.

#### 2.5.3. Conners’ Parent Rating Scales Revised

This questionnaire is utilized to evaluate potential behavioral and cognitive changes in children and adolescents aged 3 to 17 years. The areas of examination encompass hyperactivity, aggressive behavior, impulsivity, oppositional behavior, challenges in behavioral regulation, attention deficits, and modifications in working memory. Parents who complete the questionnaire should provide information about their child’s and adolescent’s behavior over the past month. Each questionnaire item has four possible responses. Scores range from 0 to 3. A score lower than 60 is not considered indicative of pathology, a score between 60 and 70 suggests pathology, and a score higher than 70 indicates pathology [35].

The questionnaire also investigates four conditions: oppositional behavior, cognitive difficulty, hyperactivity, and attention-deficit hyperactivity disorder (ADHD) index. Oppositional behavior is related to rule-breaking behaviors, problems with authority figures, and easy irritability. Cognitive difficulty concerns children and adolescents with learning problems, organization, task completion, and concentration. Hyperactivity concerns subjects with difficulty sitting for long periods and who are restless and impulsive. Finally, the CPRS-R is a screening tool to identify children and adolescents at risk for ADHD. The raw scores obtained from the questionnaire undergo standardization by calculating the T variable, enabling comparison with reference values from the general population. The profile sheet utilized for scoring and the subsequent conversion into standardized T scores takes into account gender diversity. Higher T scores indicate more pronounced behavioral difficulties within each specific category [36].

### 2.6. Statistical Analysis

Statistical analysis compared differences between the two groups of subjects (dependent variables) according to retrospective body weight status categorized as normal weight and overweight (including obesity) and allergy status (yes, and no). Independent variables (or covariate) were as follows: age at PSG recording, years; retrospective body weight status, normal or overweight; allergy status, no or yes; oAHI; ODI; minimum SpO_2_; SpO_2_ time < 90%; snoring (% TST); age at follow-up; follow-up time; and PSQ (%); physical score; emotional score; psychosocial score; total QoL; oppositional behavior (T); cognitive disorders (T); hyperactivity (T); and ADHD index (T) at follow-up.

The data present the number of subjects in each group, followed by descriptive statistics (mean, standard deviation, minimum and maximum) for each variable. The non-parametric Mann–Whitney U test was used to compare the differences between the two groups. The chi-square test was used for categorical variables and Fisher’s exact test was used if values were <5. The *p*-value was reported for each outcome measure. A *p*-value < 0.05 was considered statistically significant. Of note is that *p*-values from 0.05 to 0.1 in a study with a relatively small sample size may require more careful and cautious evaluation. Since they can lead to greater uncertainty and a higher risk of type I errors [37,38] they are discussed in the text and highlighted in green in the tables.

The results of binary logistic regression analysis of retrospective weight status (normal weight versus overweight) and allergy status (yes, and no) are presented in a table. The T column presents the t-test values for the independent variable in the model, while the S.E. column is the standard error for that variable. The Wald column presents the Wald test statistic, which is used to test the null hypothesis that the independent variable is not associated with the dependent variable. The Significance (p) column presents the test’s significance level, i.e., the probability that the observed association between the independent and the dependent variables is random. The Exp(B) column presents the odds ratio for the independent variable, i.e., the percentage increase in the odds of the dependent variable associated with a unit increase in the independent variable. Finally, the 95% CI for B (lower limit) and 95% CI for B (upper limit) columns present the 95% confidence intervals for the odds ratio of the independent variable.

The data was documented in a Microsoft^®^ Excel^®^ database compatible with Windows 11 and subjected to statistical analysis using IBM SPSS version 22.0 specifically designed for Windows operating system. (IBM Corp., 2013, Armonk, NY, USA). Figures were implemented using IBM SPSS version 22.0 for Windows (IBM Corp.).

Graphics were created by drawing on the mean scores of PSQ positivity, physical, emotional, social, academic performance, psychosocial, total QoL, oppositional behavior, cognitive disorders, hyperactivity, and ADHD index at follow-up of the children and adolescents divided by retrospective body weight category and allergy status.

## 3. Results

**Figure 2:**
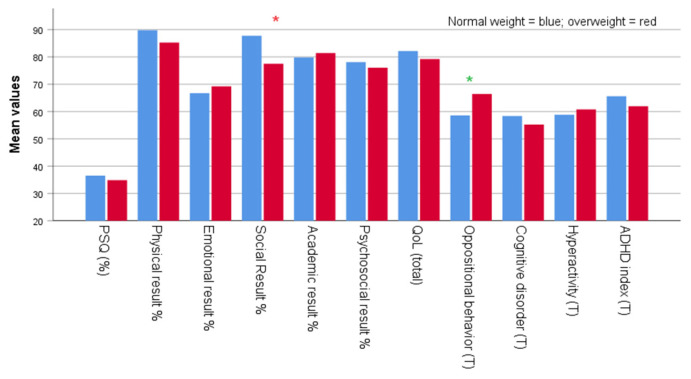
Mean scores for PSQ positivity, physical, emotional, social, academic performance, psychosocial, total QoL, oppositional behavior, cognitive disorders, hyperactivity, and ADHD index at follow-up according to retrospective body weight status. Legend: * = *p* < 0.05; * = 0.05 < *p* < 0.1.

**Figure 3:**
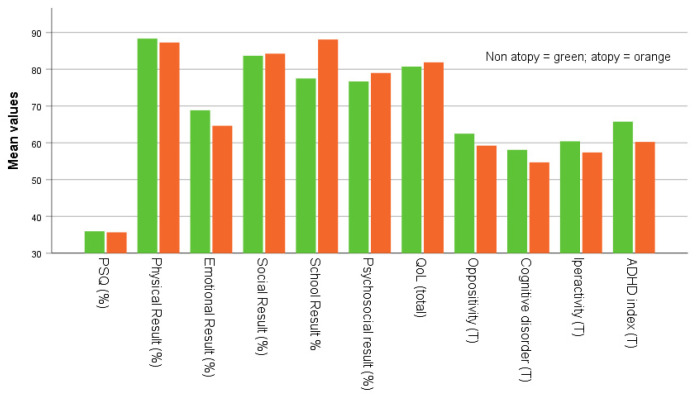
Mean PSQ positivity scores, physical, emotional, social, academic performance, psychosocial, total QoL, oppositional behavior, cognitive disorders, hyperactivity, and ADHD index at follow-up in subject with history of non-allergic and allergic status.

Table 1 summarizes the retrospective clinical data of enrolled subjects. In particular, the table shows mean, standard deviation (SD), and range for the variables age, weight, height, BMI, BMI Z-score, and BMI percentile for the four groups based on weight category (Figure 2): normal weight (*n* = 29) and overweight (*n* = 18), and on allergy status (Figure 3): non-allergic (*n* = 34) and allergic (allergic rhinitis, *n* = 13). No statistically significant differences were found for sex distribution between the four groups. There was a difference in age at PSG recording between the retrospective normal weight and the overweight status, with a higher average noted for the overweight group. There were no differences between the retrospective non-allergic and the allergic groups in age at PSG, and retrospective weight, height, BMI, BMI z-score, and BMI percentile. The retrospective higher mean body weight and height in the allergic group compared to the non-allergic group approached statistical significance (73.1 vs. 54.2) (highlighted in green).

Table 2 presents the Mann–Whitney U test of retrospective PSG data for the two groups: normal weight (*n* = 29) and overweight (*n* = 18) as mean, SD, and range. Overall, there were a few differences between the two groups: a lower mean duration of retrospective PSG data for the overweight compared to the normal weight group (9.1 ± 0.7 h vs. 9.4 ± 1.0 h.) (highlighted in green) and no differences in retrospective OSA, oAHI, and ODI, although the mean oAHI (3.9 ± 3.3 events/h vs. 5.5 ± 6.8 events/h) and the ODI (2.8 ± 2.5 events/h vs. 3.5 ± 4.9) were slightly lower in the retrospective overweight compared to the normal weight status. Furthermore, there was no significant difference in the percentage of time spent retrospectively with SpO_2_ < 90% during the eTST: lower mean minimum SpO_2_ (86.6 ± 10.1% and 89.4 ± 4.5%) in the overweight and the normal weight group, respectively. Finally, the retrospectively percentage of time spent snoring was slightly higher in the retrospective overweight compared to the normal weight status (1.7 ± 5.0% vs. 1.5 ± 4.4%). Finally, there were no significant differences between the non-allergic and the allergic group.

Table 3 presents a comparison between child’s and adolescent’s age at follow-up, length of follow-up, PSQ positivity, physical, emotional, social, and academic performance, psychosocial and total quality of life, oppositional behavior, cognitive disorders, hyperactivity, and ADHD index as mean (SD) and range for each group at follow-up. The analysis was conducted on two independent sample groups: 29 were normal weight and 18 were overweight subjects. There was a significantly higher follow-up age in the retrospectively overweight compared to the normal weight status (10.1 ± 2.5 vs. 8.2 ± 2.6) years, respectively (*p* < 0.001). There were no significant differences in PSQ positivity (%) at follow-up. The length of follow-up was slightly shorter for the retrospective overweight than the normal weight status (3.0 ± 0.9 vs. 3.2 ± 0.7 years) (highlighted in green). At follow-up, there were no statistically significant differences between the two groups for physical, emotional, psychosocial, and total quality of life outcomes at follow-up. The subjects who were normal weight, scored significantly higher on social variables compared to the subjects who were overweight (87.8 ± 16.2 vs. 77.5 ± 20.1) (*p* = 0.039), while the subjects who were overweight scored higher on oppositional behavior as measured based on the T score (66.4 ± 15.1 vs. 58.6 ± 13.4) (highlighted in green) at follow-up. There were no significant differences in cognitive disorders, hyperactivity, and ADHD index between the two groups at follow-up.

Furthermore, we found no significant differences between subjects that retrospectively declared the non-allergic and the allergic status for other variables (age and length of follow-up, PSQ positivity, physical, emotional, social, psychosocial, academic results, total QoL, oppositional behavior, cognitive disorders, hyperactivity, and ADHD index) at follow-up. This lack of difference suggests that previous respiratory allergies did not appear to affect long term quality of life or neuropsychological functions differently at follow-up in these children and adolescent.

Table 4 presents the results of a binary logistic regression analysis in which the independent variable was retrospective normal body weight (0) and overweight (1) condition. The model included two groups of variables: (1) retrospective age at PSG recording, oAHI, ODI, minimum SpO_2_ saturation, mean SpO_2_, time of SpO_2_ < 90%, and snoring (% of total sleep time); and (2) age at follow-up, follow-up length, physical, emotional, psychosocial, and total quality of life outcomes, oppositional behavior, cognitive disorders, hyperactivity, and ADHD score index at follow-up.

There was a significant and positive association between age at PSG recording and the retrospective body weight category, indicating an increased likelihood of being overweight with increasing age at PSG recording. The Exp(B) was 1.358, which means that for each year older at PSG recording, the likelihood of being obese increased by 35.8%. Furthermore, the 95% confidence interval for Exp(B) was 1.055 to 1.747, suggesting that we can be reasonably confident that the actual Exp(B) value falls within this range with 95% confidence.

The associated odds ratio for the emotional disorders at follow-up was 1.082, indicating that for each unit increase in emotional disorder at follow-up, the likelihood of being retrospectively obese increased by approximately 8.2% compared to being retrospectively normal weight. For the oppositional behavior T points at follow-up, the associated odds ratio was 1.090, indicating that for each unit increase in oppositional behavior T point at follow-up, the likelihood of being retrospectively obese increased by approximately 9% compared to the retrospective normal weight status. The associated odds ratio for the social disorder was 0.890 at follow-up, indicating that for each unit increase in social disorders at follow-up, the likelihood of being retrospectively overweight decreased by 11%. The odds ratio for the cognitive disorder T scores was 0.894 at follow-up, indicating that for each unit increase in cognitive outcomes at follow-up, the likelihood of being retrospectively overweight decreased by 10.6%. Finally, the likelihood of being retrospectively allergic approached statistical significance in children and adolescents, scoring retrospectively high on body weight and snoring (*p* = 0.090) status.

There was no significant association between emotional disorders at follow-up and retrospective allergy status (*p* = 0.070), whereas there was a significant association between allergy status and school performance at follow-up (*p* = 0.032). The odds ratio for school performance at follow-up was 1.061, indicating that for each unit increase in school performance at follow-up, the odds of retrospective allergic status increased by 6.1%. The confidence intervals of the odds ratio for both variables do not encompass 1, indicating that these data are statistically significant.

## 4. Discussion

We specifically evaluated the influence of body weight and allergy history on the mental health, cognitive development, and quality of life in children and adolescents who snore. This approach allowed us to examine the potential associations and outcomes over time in relation to snoring, body weight, and allergy status. Our study findings have clinical implications for assessing the mental health and cognitive performance at follow-up of children and adolescents who scored high on retrospective snoring and differed in body weight and allergy status. The associations between neuropsychological disorders at follow-up and retrospective body weight and allergy status may help to better understand the mechanisms underlying SDB and identify treatments that may improve the mental and cognitive health of the affected individuals. Finally, snoring can have negative impacts on mental health and cognitive development in the long term. Early detection and intervention for neuropsychological disorders is important in children and adolescents who snore. In the long term, the effects of snoring on neuropsychological disorders may vary based on previous body weight and allergy status.

No significant differences were found for baseline PSG respiratory parameters or PSQ (%) scores at follow-up between the groups scoring high on snoring (i.e., retrospectively normal weight and overweight status, and allergic and non-allergic status). The age at follow-up was significantly older for the retrospectively overweight and snoring group compared to the normal weight group. Unfortunately, we do not have anthropometric data at the follow-up. However, the age at follow-up was significantly older in the retrospectively overweight group compared to the normal weight and snoring group. This difference suggests that subjects with retrospectively overweight status and high snoring scores may be older at the follow-up assessment. The absence of anthropometric data at the follow-up precludes drawing definitive conclusions about the physical differences between the groups at that specific time point.

There were minor differences in neurocognitive scores at follow-up between the overweight subjects retrospectively scoring high on snoring (emotional and oppositional disorders) and the normal weight subjects (social and cognitive disorders). Additionally, significant differences were found between the two groups that retrospectively scored high on snoring, regardless of allergy status, in terms of school performance at follow-up, which showed a significant association with allergy status.

Children with SDB are noted to show deficits in neurocognitive performance, behavioral impairment, and school performance [39]. Previous studies report worse school performance, lower neurocognitive test scores, and behavioral abnormalities in children with SDB [9]. Children with a higher AHI were found to have more impairments than those with a lower AHI, indicating a reduction in grey matter and a dose–response impact of SDB [6].

A systematic review found a negative association between obesity and neurocognitive functions, such as executive functions, attention, visuospatial performance, and motor skills [40]. Childhood obesity has been associated with a lower ability to modulate the executive function network that supports visuospatial attention [41]. The BMI mediates the relationship between environmental degradation and reduction in global cortical and prefrontal volume, as well as the performance on cognitive tasks, such as working memory and cognitive flexibility [42]. Students with severe obesity show a delay in cognitive functions compared to those with a normal BMI. Reductions in cognitive function related to attention, memory, intelligence, and cognitive flexibility were observed in children with severe obesity [43].

Adolescents with OSAS were noted to show worse executive function and attention and were at greater risk of depression and externalizing symptoms compared to non-obese controls. Obese adolescents with OSAS may present significant neurobehavioral deficits that may persist into adulthood [44]. Adolescents with obesity and OSAS were found to have worse executive function than the normative sample. A strong association between obesity, OSAS, and cognitive impairment has been reported [45]. In addition, the severity of SDB may affect brain health and school performance in overweight children. A previous study suggested that the severity of SDB may impact school performance in overweight children but does not appear to affect brain structure [7].

High BMI and younger age at the time of testing are the two factors that may negatively affect outcomes in some domains [46]. In our study, emotional and oppositional behavior (T-scores) were significantly associated with overweight in children and adolescent scoring high on snoring. The two variables, social outcomes and cognitive disorders, were inversely associated with the condition, i.e., they were significantly associated with normal weight.

Previous studies have suggested a correlation between inhalant allergy and various neurocognitive functions in children scoring high on snoring. Multiple pediatric studies, based on surveys of parents or teachers, have found an association between SDB in childhood and aggressive behavior, impulsivity, hyperactivity, and decreased attention [39]. Inhalant allergy has emerged as one of the most critical risk factors for habitual snoring in children. It seems to increase the risk of OSA, with a significant and independent association between the severity of inhalant allergy and the severity of pediatric OSA [25].

In a cross-sectional study, the total cognitive function composite score among children who habitually snored was significantly lower. When adjusted for demographic, anthropometric, and socioeconomic characteristics, the association was substantially attenuated [47]. Our findings suggest that an increase in BMI percentile in the allergic group could constitute a risk factor for OSA. Furthermore, school performance at follow-up was significantly associated with retrospective allergy status, which could be helpful in assessing the quality of life of allergic patients in terms of their academic performance. In contrast, our study found no significant associations between emotional disorders at follow-up and retrospective allergy status.

This study has some limitations, including the small sample size, which may affect the validity and generalizability of the results. In addition, there may have been a difference in participant selection or follow-up duration between the two groups. Nonetheless, we were able to test the associations between neurobehavioral disorders and body weight, which is relevant for identifying interventions that may improve the mental and cognitive health of children and adolescents with SDB.

Various studies have examined neuropsychological and behavioral functioning in children with SDB [48,49,50] or snoring [47,51], through questionnaire-based designs [52] or before and after adenotonsillectomy [53]. Our retrospective cohort study addressed some additional key points in the current literature. Firstly, children and adolescents who scored high on snoring and had a history of overweight may develop emotional and oppositional symptoms, whereas normal weight children scoring high on snoring may exhibit more social and cognitive symptoms. Secondly, school performance at follow-up was significantly associated with a history of allergy in children and adolescents who scored high on snoring. Finally, in the long term, the effects of snoring on neuropsychological disorders may vary based on the history of overweight and allergy status.

In summary, we observed differences in neuropsychological disorders at follow-up between children and adolescents with retrospectively classified overweight status and those with normal weight who scored high on snoring. Emotional and oppositional symptoms at follow-up were associated with being overweight. Social outcomes and cognitive disorders variables at follow-up were inversely associated with retrospectively classified overweight status, meaning they were associated with normal body weight. Lastly, no differences were found in the quality of life and neuropsychological functions at follow-up between children who scored high on retrospective snoring and those with allergic rhinitis, although it is worth noting that such symptoms in academic settings were significantly associated with allergy.

## 5. Conclusions

Our study findings may have practical implications in the clinical setting. We found a correlation between age at diagnosis of SDB and obesity history in children and adolescents scoring high on snoring. Retrospective obesity status associated with snoring may become increasingly challenging to manage as affected subjects grow older. Additionally, retrospective overweight status may be at greater risk of developing emotional and oppositional symptoms, whereas normal weight status scoring high on snoring may exhibit more social and cognitive symptoms at follow-up. Furthermore, assessing the quality of life at follow-up in subjects scoring high on retrospective allergy status and snoring may be helpful, especially regarding academic performance at follow-up. These findings suggest early detection and intervention for neuropsychological disorders in children and adolescents scoring high on snoring, as snoring may negatively impact their mental health and cognitive development, though the effects may vary based on body weight and allergy status.

## Figures and Tables

**Table 1:** This table shows the descriptive statistics of clinical data collected retrospectively. It represents the mean, standard deviation, minimum and maximum for the variables of interest in the two groups of subjects who snore categorized by retrospective body weight (normal weight vs. overweight) and allergy status (non-allergic vs. allergic). Legend: BMI, body mass index. Legend: orange box = *p* < 0.05; green box = 0.05 < *p* < 0.1.

	Normal Weight (*n* = 29)		Overweight (*n* = 18)		Mann–Whitney U Test	Non-Allergic (*n* = 34)		Allergic (*n* = 13)		Mann–Whitney U Test
	Mean (SD)	Min–Max	Mean (SD)	Range	*p*-Value	Mean (SD)	Range	Mean (SD)	Range	
No. (males %)	62	-	72.2	-	0.349 [chi square]	64.7	-	69.2		0.527 [chi square]
Age at PSG (years)	5.0 (2.7)	2.0–11.1	7.1 (2.4)	3.1–13.8	0.005	5.5 (3.0)	2–13.8	6.4 (2.1)	2.9–11.0	0.183
Weight (kg)	17.8 (6.7)	6.5–39	34.9 (19.3)	17–102	<0.001	23.5 (17.0)	6.5–102	26.8 (10.3)	13–51	0.070
Height (cm)	106.8 (18.5)	62–145	123 (18.0)	93–173	0.004	110.5 (21.0)	62–173	119.4 (15.0)	90–149	0.060
BMI (kg/m^2^)	15.2 (1.5)	12.5–18.5	21.6 (4.7)	16.8–34.1	<0.001	17.5 (4.7)	12.5–34.1	18.0 (3.6)	13.3–26.7	0.385
BMI Z-score	−0.72 (1.39)	−3.80–0.80	1.9 (0.6)	1.0–2.9	<0.001	0.06 (1.79)	−3.80–2.9	0.87 (1.41)	−2.60–2.80	0.147
BMI percentile	37.5 (29.1)	0.1–80	94.6 (5.2)	84.4–99.8	<0.001	54.2 (37.4)	0.1–99.8	73.1 (29.9)	0.5–99.7	0.150

**Table 2:** Statistical analysis comparing the retrospective PSG recordings between two groups categorized by retrospective body weight (normal weight and overweight) and allergy (non-allergic and allergic, allergic rhinitis) status. The data indicate the number of subjects in each group, followed by descriptive statistics (mean, SD, and range) for the baseline variables of interest (eTST, estimated total sleep time; h, hours; OA, obstructive apnea; oAHI, obstructive apnea–hypopnea index; ODI, oxygen desaturation index; SD, standard deviation). Legend: green box = 0.05 < *p* < 0.1.

	Normal Weight (*n* = 29)		Overweight (*n* = 18)		Mann–Whitney U Test	Not-Allergic (*n* = 34)		Allergic (*n* = 13)		Mann–Whitney U Test
	Mean (SD)	Range	Mean (SD)	Range	*p*	Mean (SD)	Range	Mean (SD)	Range	
Duration of recording (h)	9.4 (1.0)	7.2–11.0	9.1 (0.7)	8.1–11.0	0.096	9.3 (0.9)	7.6–11	93.2 (0.9)	7.2–11.0	0.886
OA (Events/h)	3.5 (5.4)	0–22	2.6 (3.0)	0–10.4	0.974	3.3 (5.3)	0.0–22.0	2.8 (1.9)	0.2–7.5	0.091
oAHI (Events/h)	5.5 (6.8)	0–24.6	3.9 (3.3)	0.2–12.5	0.878	7.0 (7.1)	0.1–29.7	6.2 (2.2)	1.0–9.8	0.140
ODI (Events/h)	3.5 (4.9)	0.1–23.4	2.8 (2.5)	0.4–8.3	0.577	3.4 (4.5)	0.1–23.4	2.8 (2.7)	0.1–8.3	0.991
SpO_2_ minimum (%)	89.4 (4.5)	77–95	86.6 (10.1)	51–94	0.509	87.9 (8.1)	51–95	89.4 (4.2)	78–94	0.848
SpO_2_ < 90% (% eTST)	0.27 (0.80)	0–4.2	0.3 (0.8)	0.0–3.3	0.776	0.25 (0.74)	0.0–4.2	0.39 (0.97)	0.0 -3.3	0.670
Snoring (% eTST)	1.5 (4.4)	0.1–22.1	1.7 (5.0)	0.1–21.4	0.243	2.11 (5.3)	0.1–22.1	0.07 (0.12)	0.1–0.4	0.661

**Table 3:** The number of subjects in each group (history of normal weight versus overweight status and not allergic versus allergic status) and descriptive statistics, including mean, SD, range for the variables of interest at follow-up (questionnaires result). The Mann–Whitney U test was used to compare differences between the compared groups. Legend: orange box = *p* < 0.05; green box = 0.05 < *p* < 0.1.

	Normal Weight (*n* = 29)		Overweight (*n* = 18)		Mann–Whitney U Test	Not-Allergic		Allergic		Mann–Whitney U Test
	Mean (SD)	Range	Mean (SD)	Min–Max	*p*-Value	Mean (SD)	Range	Mean (SD)	Range	
Age at follow-up (years)	8.2 (2.6)	4.1–14.2	10.1 (2.5)	5.6–15.3	<0.001	8.7 (3.0)	4.1–15.3	9.6 (1.6)	6.7–12.8	0.191
Length follow-up (years)	3.2 (0.7)	1.7–4.5	3.0 (0.9)	1.5–4.5	0.099	3.2 (0.7)	1.5–4.5	3.1 (0.9)	1.8–4.5	0.521
PSQ (%)	36.5 (20.7)	0–77.3	34.8 (18.2)	9.1–68.2	0.775	36.0 (20.6)	0.0–77.3	35.7 (17.5)	9.1–72.7	0.962
Physical score %	89.8 (12.9)	46.9–100	85.2 (13.0)	59.4–100	0.208	88.3 (13.5)	46.9–100	87.3 (12.0)	68.8–100	0.681
Emotional score %	66.7 (15.6)	30–100	69.2 (16.9)	30–90	0.501	68.8 (15.9)	30–100	64.6 (16.5)	30–90	0.556
Social score %	87.8 (16.2)	45–100	77.5 (20.1)	35–100	0.039	83.7 (19.2)	35–100	84.2 (16.3)	55–100	0.942
Academic score %	79.8 (18.5)	40–100	81.4 (21.2)	25–100	0.601	77.5 (20.5)	25–100	88.1 (14.1)	50–100	0.116
Psychosocial score %	78.1 (13.6)	46.7–93.3	76.0 (16.6)	30–96.7	0.733	93.3 (76.7)	30–93.3	79.0 (13.7)	46.7–96.7	0.793
QoL total %	82.2 (12.5)	52.2–95.7	79.2 (14.1)	47.8–95.7	0.518	80.7 (13.7)	47.8–95.7	81.9 (11.8)	54.3–95.7	0.981
Oppositional behavior (T score)	58.6 (13.4)	38–88	66.4 (15.1)	49–98	0.082	62.5 (15.4)	38–98	59.2 (11.7)	46–88	0.584
Cognitive performance (T score)	58.3 (15.3)	41–94	55.2 (16.2)	41–95	0.346	58.1 (15.8)	41–95	54.7 (15.2)	41–94	0.575
Hyperactivity (T score)	58.8 (13.6)	39–82	60.8 (15.1)	42–84	0.677	60.4 (14.1)	39–84	57.4 (14.2)	40–83	0.497
ADHD Index (T score)	65.6 (15.6)	42–94	61.9 (13.8)	38–90	0.490	65.7 (15.0)	42–94	60.2 (14.3)	38–83	0.239

**Table 4:** Binary logistic regression analysis. The “T” column presents the t-test value for the independent variable in the model, while the “S.E.” column presents the standard error for that variable. The “Wald” column presents the Wald test statistic, which was used to test the null hypothesis that the independent variable was not associated with the dependent variable. The “*p*-value” column presents the test significance, i.e., the probability that the observed association between the independent and the dependent variables is random. The “Exp(B)” column presents the odds ratio for the independent variable, i.e., the percentage increase in the odds of the dependent variable associated with a unit increase in the independent variable. Finally, the “95% CI for B (Lower Bound)” and the “95% CI for B (Upper Bound)” columns present the 95% confidence intervals for the odds ratio of the independent variable. Legend: orange box = *p* < 0.05; green box = 0.05 < *p* < 0.1.

	Variables in the Model	T	S.E.	Wald	*p*-Value	Exp(B)	95% CI for B (Lower Limit)	95% CI for B (Upper Limit)
Dependent variable: retrospective normal weight = 0, overweight/obese = 1 status								
Retrospective age at PSG recording (years), allergy (no, yes), oAHI, ODI, minimum SpO_2_, SpO_2_ time < 90%, snoring (% TST), PSQ % at follow-up	Age at PSG recording (years)	0.306	0.129	5.633	0.018	1.358	1.055	1.747
Age at follow-up (years), follow-up time (years), retrospective allergic status (no, yes), and PSQ (%), physical performance, emotional outcomes, psychosocial outcomes, total QoL, oppositional behavior T-points, cognitive disorders T-points, hyperactivity T-points, ADHD T-point index at follow-up.	Age (years)	0.413	0.197	4.384	0.036	1.511	1.027	2.223
	Allergy (No, yes)	−1.683	0.994	2.868	0.090	0.186	0.026	1.303
	Emotional disorders	0.079	0.039	4.132	0.042	1.082	1.003	1.168
	Oppositional behavior	0.086	0.035	5.592	0.015	1.090	1.017	1.168
	Social disorders	−0.117	0.046	6.592	0.010	0.890	0.814	0.981
	Cognitive disorders	−0.112	0.052	4.587	0.032	0.894	0.806	0.991
Dependent variable: retrospective non-allergic = 0, allergic = 1 status								
Retrospective age at PSG recording (years), weight (normal, overweight), OAHI, ODI, minimum SpO_2_, SpO_2_ time < 90%, snoring (% TST), PSQ % at follow-up	None							
Age at follow-up (years), follow-up time (years), retrospective weight (normal, overweight) status, and PSQ (%), physical performance, emotional outcomes, psychosocial outcomes, total QoL, oppositional behavior (T), cognitive disorders (T), hyperactivity (T), ADHD index (T) at follow-up.	Emotional disorders	−0.052	0.029	3.278	0.070	0.950	0.888	1.004
	Academic achievement	0.060	0.028	4.594	0.032	1.061	1.005	1.121

## Data Availability

The data is unavailable due to privacy and ethical restrictions.
